# (1*R*,2*S*,4a*R*,6*S*,8*R*,8a*S*)-1-(3-Hy­droxy­propano­yl)-1,3,6,8-tetra­methyl-1,2,4a,5,6,7,8,8a-octa­hydronaphthalene-2-carb­oxy­lic acid

**DOI:** 10.1107/S241431462400885X

**Published:** 2024-09-17

**Authors:** Christo J. Botha, Gerda Fouche, Frederick P. Malan

**Affiliations:** a Department of Paraclinical Sciences, Faculty of Veterinary Science, Onderstepoort, University of Pretoria, Pretoria, South Africa; b Department of Chemistry, Faculty of Natural and Agricultural Sciences, University of Pretoria, Pretoria, South Africa; Howard University, USA

**Keywords:** crystal structure, diplodiatoxin, absolute configuration

## Abstract

The mol­ecular structure of C_18_H_28_O_4_, (+)-diplodiatoxin, is described, whereby the absolute configuration of the structure of diplodiatoxin has been confirmed by single-crystal X-ray diffraction. Diplodiatoxin crystallizes in the chiral *P*4_3_2_1_2 space group with one mol­ecule in the asymmetric unit.

## Abstract 2

The single-crystal structure elucidation of diplodiatoxin, a toxic metabolite of *Diplodia maydis*, is described.

## Graphical abstract

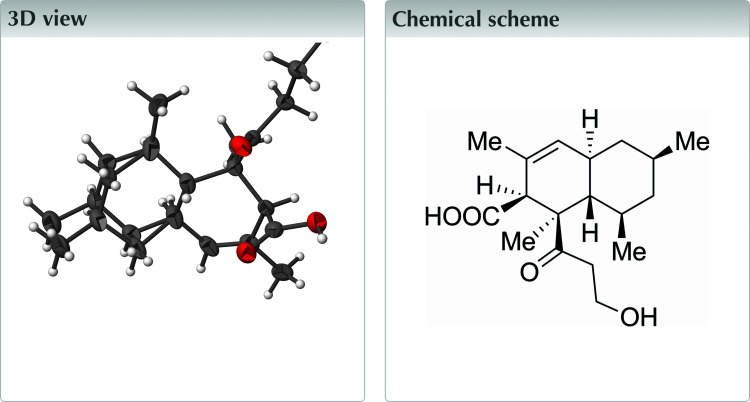

## Structure description

*Stenocarpella maydis*, an important phytopathogen of maize, is the cause of diplodiosis, a neuromuscular disease of ruminants (Masango *et al.*, 2015 ▸). Diplodiatoxin, a major metabolite isolated from *S. maydis*-infected maize cultures, contains a β-ketol side chain and a rare β,γ-unsaturated acid unit (Steyn *et al.*, 1972 ▸). Studies in ducklings (Rabie *et al.*, 1985 ▸) and rats (Rahman *et al.*, 2002 ▸) have confirmed that it induces acute toxicity and liver degeneration as well as various other toxic effects, including decreased body weight, tremors and convulsions. The cytotoxicity of three *S. maydis* metabolites (diplodiatoxin, dipmatol and diplonine) was investigated on Neuro-2a, CHO-K1 and MDBK cell lines (Masango *et al.*, 2014 ▸). Diplodiatoxin was the most cytotoxic metabolite and results obtained indicated that diplodiatoxin exerted its toxicity possibly *via* the necrotic cell death pathway.

**Figure 1:**
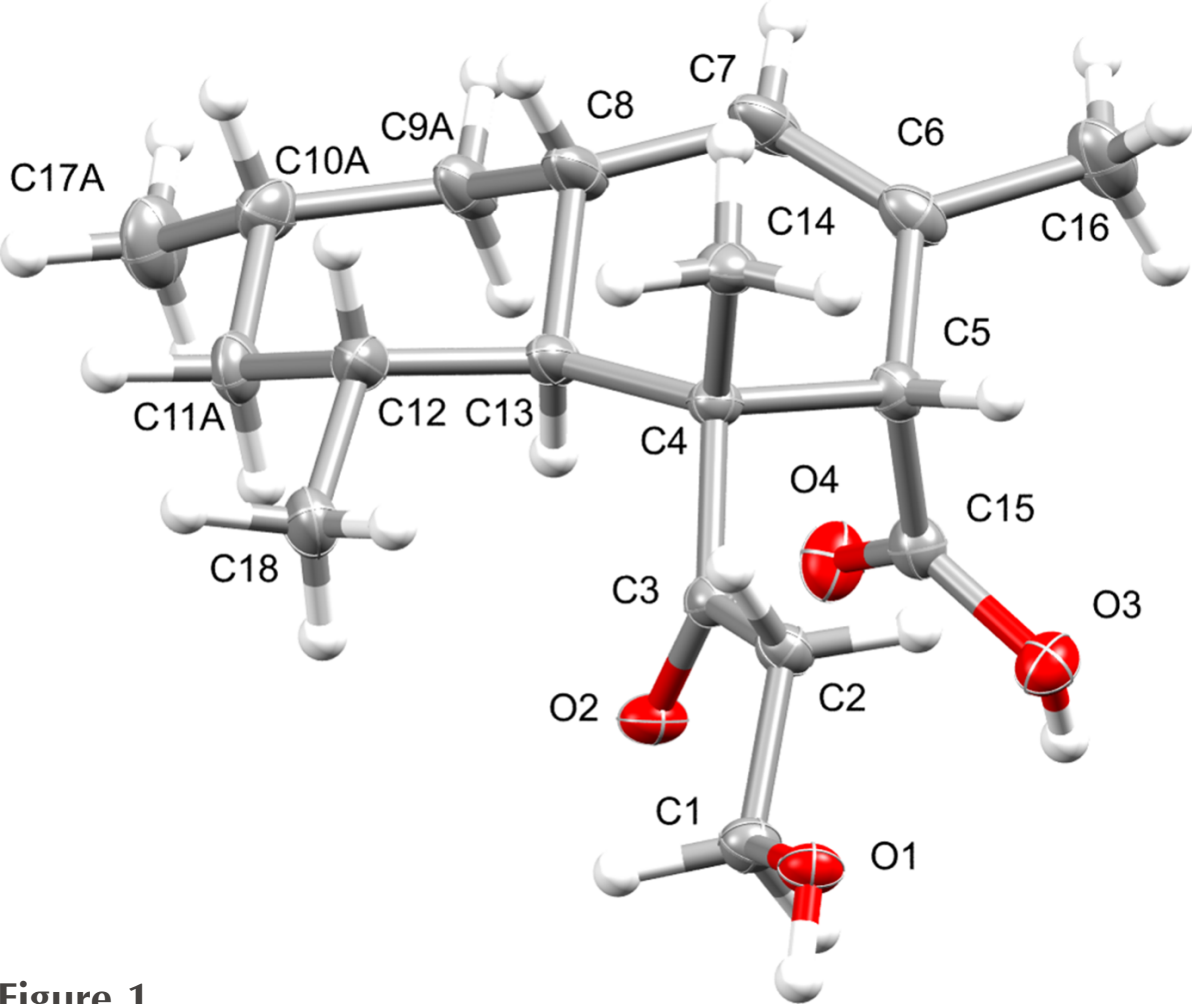
Perspective view of the mol­ecular structure of the title compound showing displacement ellipsoids at the 50% probability level. Only one disorder component is shown.

The mol­ecular structure of the title compound is shown in Fig. 1 ▸. The compound crystallizes in the chiral *P*4_3_2_1_2 space group with *Z* = 8 and *Z*′ = 1. The mol­ecule belongs to the class of phytotoxins featuring two fused six-membered carbocyclic rings and a β-ketol side chain. The relative stereochemistry of the previously determined crystal structure of (+)-diplodiatoxin at room temperature (CSD refcode DIPLOD; Kruger *et al.*, 1977 ▸) corresponds to the stereochemistry observed in this structure. However, a different space group was observed in DIPLOD (*P*4_1_2_1_2) with unit-cell parameters [*a* = 7.400 (3), *b* = 7.400 (3), *c* = 65.110 (4), volume = 3565.424 Å^3^] that differ notably due to unit-cell contraction with the corresponding parameters from this structure [*a* = 7.3410 (1), *b* = 7.3410 (1), *c* = 64.8549 (13), volume = 3495.05 (10) Å^3^]. In addition, the paper of H. D. Flack that reports the use of the Flack parameter for the first time was only published in 1983 (Flack, 1983 ▸), and therefore there existed no direct or convenient way for absolute configuration (chirality) determination of a structure using crystallographic methods alone. The South Africa-based research groups originally studying diplodiatoxin made use of ^1^H NMR methods and extensive comparisons against closely related reference compounds of which the stereochemistry was known to propose the stereochemistry of diplodiatoxin. However, in terms of the absolute configuration determination using X-ray techniques, either of the enanti­omorphous space groups *P*4_1_2_1_2 (DIPLOD) and *P*4_3_2_1_2 (this work) are plausible space groups as both exhibit the same systematic absences. However, since a single enanti­omer of a chiral compound can only crystallize in one of the enanti­omorphous space groups, and coupled with a Flack parameter of −0.02 (7), the proposal is made that the correct space group is indeed *P*4_3_2_1_2.

**Figure 2:**
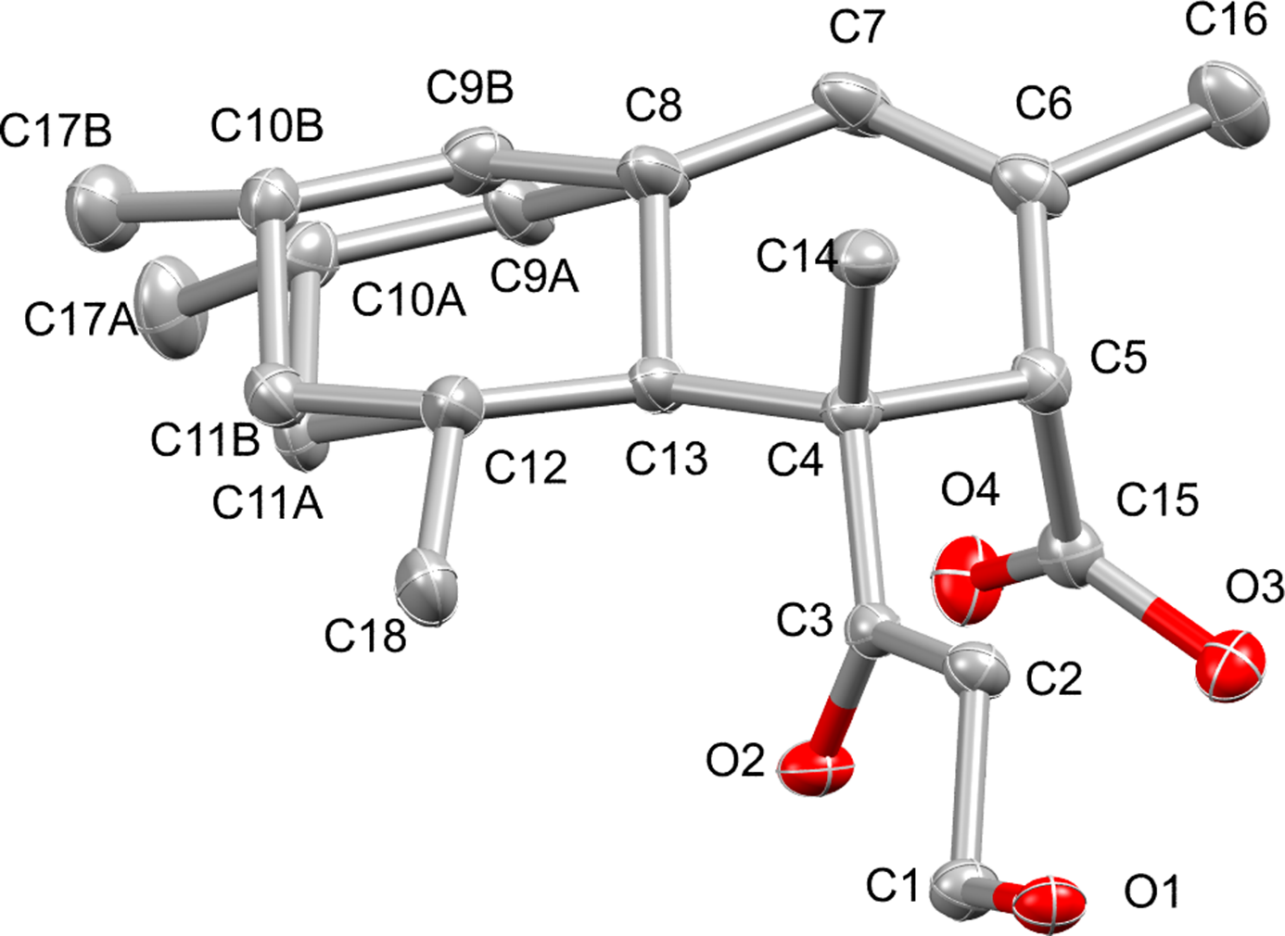
Perspective view of diplodiatoxin indicating the modelled disorder. Displacement ellipsoids are displayed at the 50% probability level. Hydrogen atoms are omitted for clarity.

**Figure 3:**
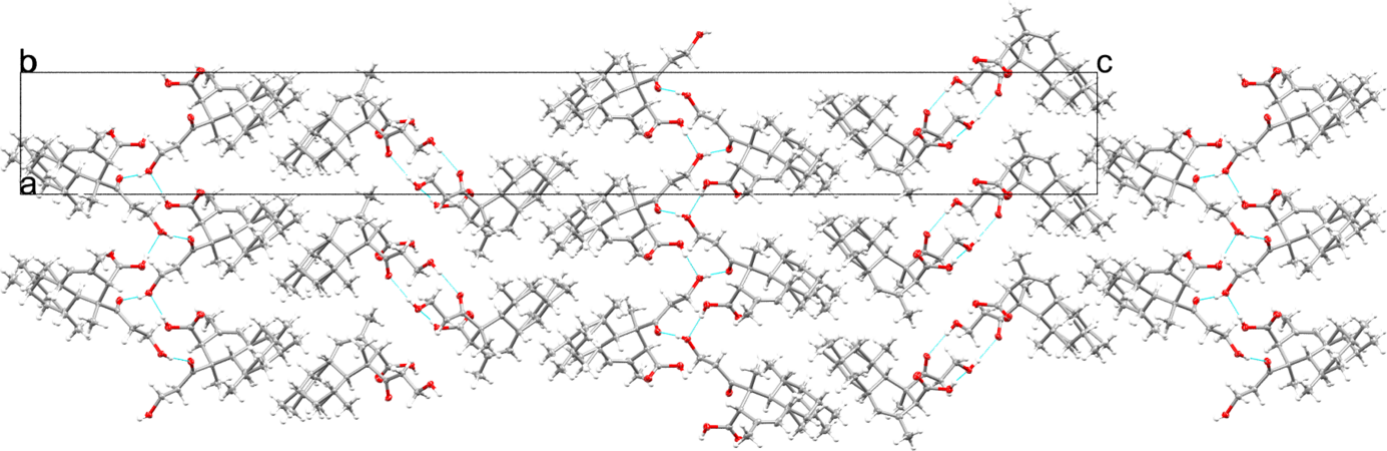
Packing diagram viewed along the *a* axis, indicating hydrogen bonds by cyan lines.

In this structure, a positional disorder of half of a chair-conformation six-membered ring has been observed, that leads to a more ‘relaxed’ chair conformation. This disorder has been modelled accordingly using free variables that refined to a 0.49:0.51 ratio (Fig. 2 ▸). All relevant bond lengths and angles observed in this structure correspond to those of DIPLOD, *i.e.* the carbonyl bond lengths of the COOH and COCH_2_ functional groups are observed to be 1.208 (3) and 1.222 (3) Å, respectively. The C—OH bond distances in CH_2_OH and COOH were found to be 1.429 (3) Å and 1.335 (3) Å, respectively. The presence of the alkene bond was also confirmed with a C=C bond length of 1.321 (4) Å (1.326 Å in DIPLOD). The supramolecular structure resulting from inter­molecular hydrogen bond inter­actions reveals corrugated layers of diplodiatoxin mol­ecules with each mol­ecule linked to four different diplodiatoxin mol­ecules *via* two strong O—H⋯O bonds of O1—H1⋯O2 (Fig. 3 ▸; Table 1 ▸).

## Synthesis and crystallization

Diplodiatoxin was isolated, purified and characterized as previously described (Botha *et al.*, 2020 ▸). Colourless single crystals suitable for X-ray diffraction was obtained by recrystallization using ethyl acetate as solvent (slow evaporation).

## Refinement

Crystal data, data collection and structure refinement details are summarized in Table 2 ▸.

## Supplementary Material

Crystal structure: contains datablock(s) I. DOI: 10.1107/S241431462400885X/bv4052sup1.cif

Structure factors: contains datablock(s) I. DOI: 10.1107/S241431462400885X/bv4052Isup2.hkl

Supporting information file. DOI: 10.1107/S241431462400885X/bv4052Isup3.cdx

Supporting information file. DOI: 10.1107/S241431462400885X/bv4052Isup4.cml

CCDC reference: 2383086

Additional supporting information:  crystallographic information; 3D view; checkCIF report

## Figures and Tables

**Table 1:** Hydrogen-bond geometry (Å, °)

*D*—H⋯*A*	*D*—H	H⋯*A*	*D*⋯*A*	*D*—H⋯*A*
C5—H5⋯O1^i^	1.00	2.60	3.571 (3)	163
O3—H3⋯O1^ii^	0.84	1.82	2.658 (2)	172
C2—H2*A*⋯O4^iii^	0.99	2.30	3.176 (3)	146
C2—H2*B*⋯O3^iv^	0.99	2.54	3.460 (3)	155
O1—H1⋯O2^v^	0.84	1.89	2.719 (2)	172

Symmetry codes: (i) 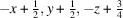; (ii) 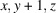; (iii) 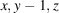; (iv) 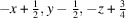; (v) 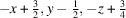.

**Table 2:** Experimental details

Crystal data	
Chemical formula	C_18_H_28_O_4_
*M*_r_	308.40
Crystal system, space group	Tetragonal, *P*4_3_2_1_2
Temperature (K)	150
*a*, *c* (Å)	7.3410 (1), 64.8549 (13)
*V* (Å^3^)	3495.05 (12)
*Z*	8
Radiation type	Cu *K*α
μ (mm^−1^)	0.65
Crystal size (mm)	0.23 × 0.21 × 0.06

Data collection	
Diffractometer	XtaLAB Synergy R, DW system, HyPix
Absorption correction	Multi-scan (*CrysAlis PRO*; Rigaku OD, 2022 ▸)
*T*_min_, *T*_max_	0.530, 1.000
No. of measured, independent and observed [*I* > 2σ(*I*)] reflections	23874, 3437, 3350
*R*_int_	0.041
(sin θ/λ)_max_ (Å^−1^)	0.617

Refinement	
*R*[*F*^2^ > 2σ(*F*^2^)], *wR*(*F*^2^), *S*	0.044, 0.097, 1.13
No. of reflections	3437
No. of parameters	243
H-atom treatment	H-atom parameters constrained
Δρ_max_, Δρ_min_ (e Å^−3^)	0.14, −0.17
Absolute structure	Flack *x* determined using 1195 quotients [(*I*^+^)−(*I*^−^)]/[(*I*^+^)+(*I*^−^)] (Parsons *et al.*, 2013 ▸)
Absolute structure parameter	−0.02 (7)

Computer programs: *CrysAlis PRO* (Rigaku OD, 2022 ▸), *SHELXT* (Sheldrick, 2015*a* ▸), *SHELXL* (Sheldrick, 2015*b* ▸) and *OLEX2* (Dolomanov *et al.*, 2009 ▸).

## full crystallographic data

### (1R,2S,4aR,6S,8R,8aS)-1-(3-Hydroxypropanoyl)-1,3,6,8-tetramethyl-1,2,4a,5,6,7,8,8a-octahydronaphthalene-2-carboxylic acid Crystal data

**Table:**

C_18_H_28_O_4_	*D*_x_ = 1.172 Mg m^−^^3^
*M**_r_* = 308.40	Cu *K*α radiation, λ = 1.54184 Å
Tetragonal, *P*4_3_2_1_2	Cell parameters from 13711 reflections
*a* = 7.3410 (1) Å	θ = 2.7–78.5°
*c* = 64.8549 (13) Å	µ = 0.65 mm^−^^1^
*V* = 3495.05 (12) Å^3^	*T* = 150 K
*Z* = 8	Plate, colourless
*F*(000) = 1344	0.23 × 0.21 × 0.06 mm

### (1R,2S,4aR,6S,8R,8aS)-1-(3-Hydroxypropanoyl)-1,3,6,8-tetramethyl-1,2,4a,5,6,7,8,8a-octahydronaphthalene-2-carboxylic acid Data collection

**Table:**

XtaLAB Synergy R, DW system, HyPix diffractometer	3437 independent reflections
Radiation source: Rotating-anode X-ray tube, Rigaku (Cu) X-ray Source	3350 reflections with *I* > 2σ(*I*)
Mirror monochromator	*R*_int_ = 0.041
Detector resolution: 10.0000 pixels mm^-1^	θ_max_ = 72.1°, θ_min_ = 2.7°
ω scans	*h* = −9→9
Absorption correction: multi-scan (CrysAlisPro; Rigaku OD, 2022)	*k* = −8→9
*T*_min_ = 0.530, *T*_max_ = 1.000	*l* = −80→80
23874 measured reflections	

### (1R,2S,4aR,6S,8R,8aS)-1-(3-Hydroxypropanoyl)-1,3,6,8-tetramethyl-1,2,4a,5,6,7,8,8a-octahydronaphthalene-2-carboxylic acid Refinement

**Table:**

Refinement on *F*^2^	Hydrogen site location: inferred from neighbouring sites
Least-squares matrix: full	H-atom parameters constrained
*R*[*F*^2^ > 2σ(*F*^2^)] = 0.044	*w* = 1/[σ^2^(*F*_o_^2^) + (0.0224*P*)^2^ + 1.8437*P*] where *P* = (*F*_o_^2^ + 2*F*_c_^2^)/3
*wR*(*F*^2^) = 0.097	(Δ/σ)_max_ = 0.001
*S* = 1.13	Δρ_max_ = 0.14 e Å^−^^3^
3437 reflections	Δρ_min_ = −0.17 e Å^−^^3^
243 parameters	Absolute structure: Flack *x* determined using 1195 quotients [(*I*^+^)-(*I*^-^)]/[(*I*^+^)+(*I*^-^)] (Parsons *et al.*, 2013)
0 restraints	Absolute structure parameter: −0.02 (7)
Primary atom site location: dual	

### (1R,2S,4aR,6S,8R,8aS)-1-(3-Hydroxypropanoyl)-1,3,6,8-tetramethyl-1,2,4a,5,6,7,8,8a-octahydronaphthalene-2-carboxylic acid Special details

**Table:**

Geometry. All esds (except the esd in the dihedral angle between two l.s. planes) are estimated using the full covariance matrix. The cell esds are taken into account individually in the estimation of esds in distances, angles and torsion angles; correlations between esds in cell parameters are only used when they are defined by crystal symmetry. An approximate (isotropic) treatment of cell esds is used for estimating esds involving l.s. planes.
Refinement. The structure was solved by direct methods with the SHELXTS-2016 program, refined using the *SHELXL2016* algorithm, all using the *OLEX2* interface. All H atoms were placed in geometrically idealized positions and constrained to ride on their parent atoms. The highest calculated residual electron density is 0.10 e.Å^-3^ at 1.07 Å from C16, which is insignificant in this case.

### (1R,2S,4aR,6S,8R,8aS)-1-(3-Hydroxypropanoyl)-1,3,6,8-tetramethyl-1,2,4a,5,6,7,8,8a-octahydronaphthalene-2-carboxylic acid Fractional atomic coordinates and isotropic or equivalent isotropic displacement parameters (Å^2^)

**Table:**

	*x*	*y*	*z*	*U*_iso_*/*U*_eq_	Occ. (<1)
C15	0.4129 (4)	0.9084 (3)	0.34201 (4)	0.0332 (5)	
C14	0.2579 (3)	0.4534 (4)	0.31694 (4)	0.0350 (6)	
H14A	0.1772	0.4962	0.3059	0.053*	
H14B	0.1852	0.4234	0.3292	0.053*	
H14C	0.3234	0.3446	0.3123	0.053*	
C13	0.5014 (3)	0.6691 (4)	0.30311 (4)	0.0343 (6)	
H13	0.5875	0.7661	0.3078	0.041*	
C8	0.3723 (4)	0.7573 (5)	0.28718 (4)	0.0468 (8)	
H8A	0.3235	0.6632	0.2775	0.056*	0.494 (10)
H8B	0.3082	0.6503	0.2810	0.056*	0.506 (10)
C7	0.2209 (4)	0.8661 (4)	0.29625 (4)	0.0446 (7)	
H7	0.1513	0.9392	0.2871	0.054*	
C6	0.1752 (4)	0.8697 (4)	0.31595 (4)	0.0372 (6)	
C5	0.2884 (3)	0.7690 (3)	0.33183 (4)	0.0298 (5)	
H5	0.2038	0.7207	0.3426	0.036*	
O4	0.5103 (3)	1.0133 (3)	0.33280 (3)	0.0434 (5)	
C4	0.3950 (3)	0.6032 (3)	0.32235 (3)	0.0271 (5)	
O3	0.3983 (3)	0.9074 (3)	0.36252 (2)	0.0382 (4)	
H3	0.4571	0.9954	0.3674	0.057*	
C3	0.5263 (3)	0.5462 (3)	0.33960 (3)	0.0260 (5)	
C18	0.7363 (4)	0.3999 (5)	0.30382 (4)	0.0468 (7)	
H18A	0.8232	0.4724	0.3118	0.070*	
H18B	0.8028	0.3185	0.2945	0.070*	
H18C	0.6608	0.3275	0.3132	0.070*	
C16	0.0178 (4)	0.9783 (4)	0.32428 (5)	0.0506 (8)	
H16A	−0.0328	1.0548	0.3133	0.076*	
H16B	0.0602	1.0558	0.3356	0.076*	
H16C	−0.0765	0.8952	0.3294	0.076*	
O2	0.6696 (2)	0.6285 (2)	0.34185 (2)	0.0344 (4)	
C2	0.4710 (3)	0.3998 (3)	0.35434 (3)	0.0295 (5)	
H2A	0.4303	0.2921	0.3464	0.035*	
H2B	0.3661	0.4435	0.3626	0.035*	
O1	0.5583 (2)	0.1903 (2)	0.38058 (2)	0.0304 (4)	
H1	0.6375	0.1612	0.3893	0.046*	
C1	0.6215 (3)	0.3425 (4)	0.36885 (4)	0.0323 (5)	
H1A	0.7315	0.3083	0.3609	0.039*	
H1B	0.6534	0.4445	0.3782	0.039*	
C12	0.6150 (4)	0.5266 (5)	0.29124 (4)	0.0446 (7)	
H12	0.5335	0.4532	0.2821	0.054*	0.494 (10)
H12A	0.5228	0.4445	0.2847	0.054*	0.506 (10)
C9A	0.4987 (12)	0.9074 (14)	0.27458 (14)	0.042 (2)	0.494 (10)
H9AA	0.4225	0.9794	0.2650	0.051*	0.494 (10)
H9AB	0.5584	0.9919	0.2844	0.051*	0.494 (10)
C10A	0.6394 (9)	0.7995 (13)	0.26283 (10)	0.0426 (19)	0.494 (10)
H10A	0.5750	0.7265	0.2521	0.051*	0.494 (10)
C11A	0.7432 (14)	0.6671 (14)	0.27687 (17)	0.040 (2)	0.494 (10)
H11A	0.8215	0.7393	0.2862	0.048*	0.494 (10)
H11B	0.8246	0.5920	0.2681	0.048*	0.494 (10)
C17A	0.7749 (10)	0.9272 (11)	0.25190 (12)	0.058 (2)	0.494 (10)
H17A	0.7085	1.0082	0.2425	0.087*	0.494 (10)
H17B	0.8625	0.8545	0.2440	0.087*	0.494 (10)
H17C	0.8400	1.0000	0.2622	0.087*	0.494 (10)
C9B	0.4775 (13)	0.8310 (12)	0.26980 (13)	0.0384 (19)	0.506 (10)
H9BA	0.5492	0.9359	0.2749	0.046*	0.506 (10)
H9BB	0.3902	0.8787	0.2595	0.046*	0.506 (10)
C10B	0.6078 (9)	0.7018 (11)	0.25875 (9)	0.0357 (15)	0.506 (10)
H10B	0.5316	0.6119	0.2510	0.043*	0.506 (10)
C11B	0.7214 (13)	0.5943 (13)	0.27430 (16)	0.0360 (19)	0.506 (10)
H11C	0.7799	0.4904	0.2672	0.043*	0.506 (10)
H11D	0.8192	0.6739	0.2797	0.043*	0.506 (10)
C17B	0.7255 (9)	0.8015 (11)	0.24295 (10)	0.053 (2)	0.506 (10)
H17D	0.6476	0.8761	0.2340	0.079*	0.506 (10)
H17E	0.7916	0.7124	0.2345	0.079*	0.506 (10)
H17F	0.8128	0.8800	0.2501	0.079*	0.506 (10)

### (1R,2S,4aR,6S,8R,8aS)-1-(3-Hydroxypropanoyl)-1,3,6,8-tetramethyl-1,2,4a,5,6,7,8,8a-octahydronaphthalene-2-carboxylic acid Atomic displacement parameters (Å^2^)

**Table:**

	*U*^11^	*U*^22^	*U*^33^	*U*^12^	*U*^13^	*U*^23^
C15	0.0350 (14)	0.0275 (12)	0.0369 (12)	0.0021 (10)	0.0031 (11)	0.0020 (10)
C14	0.0293 (13)	0.0416 (15)	0.0342 (12)	−0.0039 (11)	−0.0065 (10)	−0.0013 (11)
C13	0.0253 (13)	0.0513 (16)	0.0263 (11)	0.0011 (11)	−0.0011 (10)	0.0081 (11)
C8	0.0271 (14)	0.080 (2)	0.0329 (13)	0.0079 (14)	0.0001 (11)	0.0196 (14)
C7	0.0273 (14)	0.061 (2)	0.0454 (15)	0.0040 (13)	−0.0018 (12)	0.0273 (14)
C6	0.0277 (13)	0.0373 (15)	0.0466 (14)	0.0001 (11)	−0.0003 (11)	0.0141 (12)
C5	0.0281 (12)	0.0295 (13)	0.0318 (11)	−0.0005 (10)	0.0031 (10)	0.0053 (10)
O4	0.0506 (12)	0.0335 (10)	0.0461 (10)	−0.0104 (9)	0.0129 (9)	0.0014 (8)
C4	0.0250 (11)	0.0307 (12)	0.0255 (10)	−0.0010 (10)	−0.0023 (9)	0.0028 (9)
O3	0.0428 (11)	0.0368 (10)	0.0349 (8)	−0.0075 (8)	0.0019 (8)	−0.0019 (8)
C3	0.0250 (12)	0.0271 (12)	0.0260 (10)	0.0024 (9)	−0.0013 (9)	−0.0024 (9)
C18	0.0399 (16)	0.0577 (19)	0.0429 (15)	0.0137 (14)	0.0068 (12)	−0.0039 (14)
C16	0.0451 (17)	0.0456 (17)	0.0612 (17)	0.0154 (14)	0.0062 (14)	0.0178 (14)
O2	0.0322 (10)	0.0385 (10)	0.0326 (8)	−0.0072 (8)	−0.0088 (7)	0.0060 (7)
C2	0.0280 (12)	0.0284 (12)	0.0322 (11)	0.0021 (10)	−0.0022 (10)	0.0041 (10)
O1	0.0325 (9)	0.0288 (9)	0.0300 (8)	−0.0001 (7)	−0.0063 (7)	0.0067 (7)
C1	0.0320 (13)	0.0335 (13)	0.0315 (11)	−0.0016 (10)	−0.0024 (10)	0.0057 (10)
C12	0.0329 (14)	0.072 (2)	0.0293 (12)	0.0094 (14)	0.0001 (11)	−0.0018 (13)
C9A	0.027 (3)	0.060 (6)	0.039 (4)	0.002 (4)	0.006 (3)	0.023 (4)
C10A	0.042 (4)	0.055 (5)	0.030 (3)	−0.003 (3)	0.003 (3)	0.007 (3)
C11A	0.034 (4)	0.048 (6)	0.037 (4)	0.000 (4)	0.015 (3)	0.004 (4)
C17A	0.058 (5)	0.060 (5)	0.056 (4)	0.001 (4)	0.024 (3)	0.021 (4)
C9B	0.043 (4)	0.040 (5)	0.033 (3)	0.001 (4)	−0.003 (3)	0.009 (3)
C10B	0.037 (3)	0.040 (4)	0.029 (3)	0.001 (3)	0.005 (2)	0.003 (3)
C11B	0.028 (4)	0.041 (5)	0.038 (4)	0.003 (4)	0.002 (3)	0.001 (4)
C17B	0.049 (4)	0.064 (5)	0.045 (3)	−0.001 (3)	0.010 (3)	0.014 (3)

### (1R,2S,4aR,6S,8R,8aS)-1-(3-Hydroxypropanoyl)-1,3,6,8-tetramethyl-1,2,4a,5,6,7,8,8a-octahydronaphthalene-2-carboxylic acid Geometric parameters (Å, º)

**Table:**

C15—C5	1.523 (4)	C2—H2A	0.9900
C15—O4	1.208 (3)	C2—H2B	0.9900
C15—O3	1.335 (3)	C2—C1	1.511 (3)
C14—H14A	0.9800	O1—H1	0.8400
C14—H14B	0.9800	O1—C1	1.429 (3)
C14—H14C	0.9800	C1—H1A	0.9900
C14—C4	1.532 (3)	C1—H1B	0.9900
C13—H13	1.0000	C12—H12	1.0000
C13—C8	1.544 (3)	C12—H12A	1.0000
C13—C4	1.550 (3)	C12—C11A	1.679 (11)
C13—C12	1.543 (4)	C12—C11B	1.437 (11)
C8—H8A	1.0000	C9A—H9AA	0.9900
C8—H8B	1.0000	C9A—H9AB	0.9900
C8—C7	1.490 (4)	C9A—C10A	1.509 (11)
C8—C9A	1.656 (9)	C10A—H10A	1.0000
C8—C9B	1.469 (9)	C10A—C11A	1.534 (13)
C7—H7	0.9500	C10A—C17A	1.539 (9)
C7—C6	1.321 (4)	C11A—H11A	0.9900
C6—C5	1.516 (3)	C11A—H11B	0.9900
C6—C16	1.504 (4)	C17A—H17A	0.9800
C5—H5	1.0000	C17A—H17B	0.9800
C5—C4	1.573 (3)	C17A—H17C	0.9800
C4—C3	1.535 (3)	C9B—H9BA	0.9900
O3—H3	0.8400	C9B—H9BB	0.9900
C3—O2	1.222 (3)	C9B—C10B	1.526 (10)
C3—C2	1.494 (3)	C10B—H10B	1.0000
C18—H18A	0.9800	C10B—C11B	1.528 (12)
C18—H18B	0.9800	C10B—C17B	1.527 (8)
C18—H18C	0.9800	C11B—H11C	0.9900
C18—C12	1.524 (4)	C11B—H11D	0.9900
C16—H16A	0.9800	C17B—H17D	0.9800
C16—H16B	0.9800	C17B—H17E	0.9800
C16—H16C	0.9800	C17B—H17F	0.9800
			
O4—C15—C5	124.7 (2)	C1—O1—H1	109.5
O4—C15—O3	122.9 (2)	C2—C1—H1A	110.1
O3—C15—C5	112.3 (2)	C2—C1—H1B	110.1
H14A—C14—H14B	109.5	O1—C1—C2	108.16 (19)
H14A—C14—H14C	109.5	O1—C1—H1A	110.1
H14B—C14—H14C	109.5	O1—C1—H1B	110.1
C4—C14—H14A	109.5	H1A—C1—H1B	108.4
C4—C14—H14B	109.5	C13—C12—H12	109.7
C4—C14—H14C	109.5	C13—C12—H12A	104.7
C8—C13—H13	107.1	C13—C12—C11A	99.4 (4)
C8—C13—C4	111.1 (2)	C18—C12—C13	117.5 (2)
C4—C13—H13	107.1	C18—C12—H12	109.7
C12—C13—H13	107.1	C18—C12—H12A	104.7
C12—C13—C8	106.4 (2)	C18—C12—C11A	110.1 (4)
C12—C13—C4	117.5 (2)	C11A—C12—H12	109.7
C13—C8—H8A	110.5	C11B—C12—C13	116.1 (5)
C13—C8—H8B	103.2	C11B—C12—C18	107.6 (5)
C13—C8—C9A	105.4 (4)	C11B—C12—H12A	104.7
C7—C8—C13	114.7 (2)	C8—C9A—H9AA	110.4
C7—C8—H8A	110.5	C8—C9A—H9AB	110.4
C7—C8—H8B	103.2	H9AA—C9A—H9AB	108.6
C7—C8—C9A	104.8 (4)	C10A—C9A—C8	106.5 (6)
C9A—C8—H8A	110.5	C10A—C9A—H9AA	110.4
C9B—C8—C13	110.2 (4)	C10A—C9A—H9AB	110.4
C9B—C8—H8B	103.2	C9A—C10A—H10A	108.1
C9B—C8—C7	119.8 (4)	C9A—C10A—C11A	111.9 (6)
C8—C7—H7	117.2	C9A—C10A—C17A	110.8 (6)
C6—C7—C8	125.6 (2)	C11A—C10A—H10A	108.1
C6—C7—H7	117.2	C11A—C10A—C17A	109.8 (6)
C7—C6—C5	120.5 (2)	C17A—C10A—H10A	108.1
C7—C6—C16	123.6 (2)	C12—C11A—H11A	108.3
C16—C6—C5	115.8 (2)	C12—C11A—H11B	108.3
C15—C5—H5	107.9	C10A—C11A—C12	116.1 (7)
C15—C5—C4	113.0 (2)	C10A—C11A—H11A	108.3
C6—C5—C15	107.2 (2)	C10A—C11A—H11B	108.3
C6—C5—H5	107.9	H11A—C11A—H11B	107.4
C6—C5—C4	112.6 (2)	C10A—C17A—H17A	109.5
C4—C5—H5	107.9	C10A—C17A—H17B	109.5
C14—C4—C13	111.80 (19)	C10A—C17A—H17C	109.5
C14—C4—C5	108.56 (19)	H17A—C17A—H17B	109.5
C14—C4—C3	112.6 (2)	H17A—C17A—H17C	109.5
C13—C4—C5	108.91 (19)	H17B—C17A—H17C	109.5
C3—C4—C13	110.81 (19)	C8—C9B—H9BA	107.9
C3—C4—C5	103.80 (17)	C8—C9B—H9BB	107.9
C15—O3—H3	109.5	C8—C9B—C10B	117.4 (6)
O2—C3—C4	119.5 (2)	H9BA—C9B—H9BB	107.2
O2—C3—C2	120.9 (2)	C10B—C9B—H9BA	107.9
C2—C3—C4	119.5 (2)	C10B—C9B—H9BB	107.9
H18A—C18—H18B	109.5	C9B—C10B—H10B	107.2
H18A—C18—H18C	109.5	C9B—C10B—C11B	110.7 (6)
H18B—C18—H18C	109.5	C9B—C10B—C17B	111.8 (6)
C12—C18—H18A	109.5	C11B—C10B—H10B	107.2
C12—C18—H18B	109.5	C17B—C10B—H10B	107.2
C12—C18—H18C	109.5	C17B—C10B—C11B	112.4 (6)
C6—C16—H16A	109.5	C12—C11B—C10B	112.7 (7)
C6—C16—H16B	109.5	C12—C11B—H11C	109.0
C6—C16—H16C	109.5	C12—C11B—H11D	109.0
H16A—C16—H16B	109.5	C10B—C11B—H11C	109.0
H16A—C16—H16C	109.5	C10B—C11B—H11D	109.0
H16B—C16—H16C	109.5	H11C—C11B—H11D	107.8
C3—C2—H2A	108.9	C10B—C17B—H17D	109.5
C3—C2—H2B	108.9	C10B—C17B—H17E	109.5
C3—C2—C1	113.6 (2)	C10B—C17B—H17F	109.5
H2A—C2—H2B	107.7	H17D—C17B—H17E	109.5
C1—C2—H2A	108.9	H17D—C17B—H17F	109.5
C1—C2—H2B	108.9	H17E—C17B—H17F	109.5
			
C15—C5—C4—C14	167.55 (19)	O4—C15—C5—C6	−51.3 (3)
C15—C5—C4—C13	−70.5 (2)	O4—C15—C5—C4	73.4 (3)
C15—C5—C4—C3	47.6 (2)	C4—C13—C8—C7	37.0 (4)
C14—C4—C3—O2	160.9 (2)	C4—C13—C8—C9A	151.7 (4)
C14—C4—C3—C2	−23.5 (3)	C4—C13—C8—C9B	175.8 (4)
C13—C8—C7—C6	−10.5 (5)	C4—C13—C12—C18	−46.6 (4)
C13—C8—C9A—C10A	65.9 (6)	C4—C13—C12—C11A	−165.3 (4)
C13—C8—C9B—C10B	54.0 (7)	C4—C13—C12—C11B	−176.1 (5)
C13—C4—C3—O2	34.8 (3)	C4—C3—C2—C1	173.2 (2)
C13—C4—C3—C2	−149.6 (2)	O3—C15—C5—C6	126.3 (2)
C13—C12—C11A—C10A	−57.7 (7)	O3—C15—C5—C4	−109.0 (2)
C13—C12—C11B—C10B	−54.1 (7)	C3—C2—C1—O1	−174.03 (19)
C8—C13—C4—C14	63.3 (3)	C18—C12—C11A—C10A	178.3 (6)
C8—C13—C4—C5	−56.7 (3)	C18—C12—C11B—C10B	171.8 (5)
C8—C13—C4—C3	−170.3 (2)	C16—C6—C5—C15	−78.0 (3)
C8—C13—C12—C18	−171.8 (3)	C16—C6—C5—C4	157.1 (2)
C8—C13—C12—C11A	69.5 (5)	O2—C3—C2—C1	−11.3 (3)
C8—C13—C12—C11B	58.7 (5)	C12—C13—C8—C7	166.0 (3)
C8—C7—C6—C5	4.9 (5)	C12—C13—C8—C9A	−79.3 (5)
C8—C7—C6—C16	−178.4 (3)	C12—C13—C8—C9B	−55.2 (5)
C8—C9A—C10A—C11A	−52.5 (8)	C12—C13—C4—C14	−59.5 (3)
C8—C9A—C10A—C17A	−175.4 (5)	C12—C13—C4—C5	−179.5 (2)
C8—C9B—C10B—C11B	−46.5 (9)	C12—C13—C4—C3	66.9 (3)
C8—C9B—C10B—C17B	−172.6 (6)	C9A—C8—C7—C6	−125.6 (5)
C7—C8—C9A—C10A	−172.7 (5)	C9A—C8—C9B—C10B	136 (2)
C7—C8—C9B—C10B	−169.6 (5)	C9A—C10A—C11A—C12	52.6 (9)
C7—C6—C5—C15	99.0 (3)	C11A—C12—C11B—C10B	−88 (2)
C7—C6—C5—C4	−25.8 (4)	C17A—C10A—C11A—C12	176.0 (6)
C6—C5—C4—C14	−70.8 (2)	C9B—C8—C7—C6	−145.1 (5)
C6—C5—C4—C13	51.2 (3)	C9B—C8—C9A—C10A	−39.5 (13)
C6—C5—C4—C3	169.3 (2)	C9B—C10B—C11B—C12	44.0 (8)
C5—C4—C3—O2	−81.9 (3)	C11B—C12—C11A—C10A	92 (2)
C5—C4—C3—C2	93.6 (2)	C17B—C10B—C11B—C12	169.8 (6)

### (1R,2S,4aR,6S,8R,8aS)-1-(3-Hydroxypropanoyl)-1,3,6,8-tetramethyl-1,2,4a,5,6,7,8,8a-octahydronaphthalene-2-carboxylic acid Hydrogen-bond geometry (Å, º)

**Table:**

*D*—H···*A*	*D*—H	H···*A*	*D*···*A*	*D*—H···*A*
C5—H5···O1^i^	1.00	2.60	3.571 (3)	163
O3—H3···O1^ii^	0.84	1.82	2.658 (2)	172
C2—H2*A*···O4^iii^	0.99	2.30	3.176 (3)	146
C2—H2*B*···O3^iv^	0.99	2.54	3.460 (3)	155
O1—H1···O2^v^	0.84	1.89	2.719 (2)	172

Symmetry codes: (i) −*x*+1/2, *y*+1/2, −*z*+3/4; (ii) *x*, *y*+1, *z*; (iii) *x*, *y*−1, *z*; (iv) −*x*+1/2, *y*−1/2, −*z*+3/4; (v) −*x*+3/2, *y*−1/2, −*z*+3/4.
